# The role of research competence as an influencing factor for the careers of young academics. Findings and implications from studies on doctorates in medicine and life sciences in Germany

**DOI:** 10.3205/zma001652

**Published:** 2023-11-15

**Authors:** Nurith Epstein, Julia Eberle, Julia Meuleners, Daniel Lachmann, Sonja Heuser, Stefan Herzig, Birgit Neuhaus, Martin R. Fischer

**Affiliations:** 1 LMU Hospital, LMU Munich, Institute of Medical Education, Munich, Germany; 2 Ruhr-University Bochum, Bochum, Germany; 3 LMU Munich, Faculty of Biology, Biology Education, Munich, Germany; 4 TH Cologne, Cologne, Germany

**Keywords:** academic research careers, structured doctoral programs, junior researchers, female researchers

## Abstract

**Background:**

When viewed internationally, Germany boasts a high rate of doctoral candidates. Fields such as medicine and life sciences have a notably high proportion of doctoral students, a trend rooted in historical factors. Despite this, comprehensive empirical studies concerning the doctoral phase and early-career researchers, especially in relation to the rise of structured doctoral programmes, have only recently gained traction.

**Methods:**

We present findings from a project investigating young scientists in medicine and life sciences. Postdoctoral graduates from these disciplines were examined both quantitatively and qualitatively within the E-Prom projects, emphasizing the primary domain of research.

**Results:**

Our analysis indicates some benefits of structured doctoral programmes over traditional individual doctorates. However, the disparities between these doctoral approaches are less pronounced than anticipated. We also identified discrepancies between the programme descriptions and their actual execution. Integration into the scientific community and research-related self-efficacy are potential indicators of publication output and inclination towards a scientific career. Physicians exhibited lower research-related self-efficacy and a lesser tendency towards a scientific career than biologists. Notably, we found gender disparities disadvantaging female graduates, with these disparities being more marked in medicine.

**Conclusions:**

There is evidence to suggest that official representations of structured doctoral programmes do not always align with their practical applications, limiting their potential effectiveness. Therefore, resources should be allocated to ensure the consistent execution of these programmes. Given the empirical evidence supporting the benefits of community integration for junior researchers, efforts should be made to facilitate their networking. Additionally, our findings emphasize the necessity of providing enhanced support for young female scientists.

## Zusammenfassung

**Hintergrund:**

Im internationalen Vergleich verfügt Deutschland über eine insgesamt hohe Promotionsquote. In bestimmten Fächern, wie Medizin und Lebenswissenschaften, ist der Anteil an Promovierenden aus historischen Gründen besonders hoch. Empirische Ergebnisse über die Promotionsphase und den wissenschaftlichen Nachwuchs, auch im Hinblick auf die Zunahme von strukturierten Promotionsprogrammen, wurden erst kürzlich vermehrt produziert.

**Methoden:**

Im Folgenden berichten wir über ein Projekt zum wissenschaftlichen Nachwuchs in Medizin und Lebenswissenschaften. Promovierte Mediziner und Biologen wurden im Rahmen der E-Prom-Projekte sowohl quantitativ als auch qualitativ befragt, wobei der Fokus auf dem zentralen Teilbereich der Forschung lag.

**Ergebnisse:**

Unsere Analysen zeigen gewisse Vorteile der strukturierten Promotion gegenüber der Individualpromotion, dennoch sind die Unterschiede zwischen den Promotionsformen geringer als erwartet. Zudem zeigen sich Abweichungen zwischen Programmbeschreibungen und deren Umsetzung. Die Eingebundenheit in die Wissenschaftsgemeinschaft und die forschungsbezogene Selbstwirksamkeitserwartung zeigen sich als mögliche Prädiktoren für die Publikationsoutput und die wissenschaftliche Karriere-Intention. Mediziner weisen eine geringere forschungsbezogene Selbstwirksamkeitserwartung und wissenschaftliche Karriereintention auf als Biologen. Zudem zeigten sich Geschlechterunterschiede zu Ungunsten der Absolventinnen; diese waren in der Medizin stärker ausgeprägt.

**Schlussfolgerungen:**

Offizielle Angaben zur Ausgestaltung strukturierter Promotionsprogramme weichen teilweise von deren Umsetzung ab, sodass deren Potenzial nicht optimal genutzt wird. So sollten auch Ressourcen für die Überprüfung der Programmumsetzung aufgewendet werden. Aufgrund der vielfachen empirischen Ergebnisse zum positiven Einfluss der Eingebundenheit in die Wissenschaftsgemeinschaft sollten Nachwuchswissenschaftlerinnen und -wissenschaftler beim Netzwerken besonders unterstützt werden. Im Weiteren unterstreichen unsere Ergebnisse die Notwendigkeit, Nachwuchswissenschaftlerinnen besonders zu fördern.

## Introduction

In recent years, the challenges and prospects of young academics have become central topics in education policy debates. Two primary catalysts for these discussions have been the precarious employment conditions facing many young academics and the prevalent model of individual doctorates in Germany. Despite the dominance of the individual doctorate model, there has been a growing emphasis on structured doctoral programmes. Such programmes are already the norm in countries like the USA, designed to provide superior training and streamline the selection process for budding academics. Contrasting with the conventional individually-tailored doctorates, structured doctoral programmes aim to offer a more equitable and efficient recruitment process for doctoral candidates through formalized selection procedures. These programmes also aspire to reduce the duration and enhance the outcomes of doctoral studies. Key features include multi-faceted formal supervision, the establishment of explicit goals, and fostering organized interactions with peers within the programme.

In addition to the interdisciplinary discussion about the next generation of scientists, there is also the discussion about the doctorate in medicine in Germany: unlike usual, most work on the doctorate is done while studying. Moreover, the doctorate in medicine represents the first independent scientific work, whereas in other subjects it is usually preceded by bachelor's, master's and other theses. The quality of medical doctoral theses has been questioned for some time [3], [16]. 

## Project description

As part of the BMBF funding line “Research on Young Scientists” [https://www.bmbf.de/bmbf/de/forschung/wissenschaftlicher-nachwuchs/forschung-zum-wissenschaftlichen-nachwuchs/forschung-zum-wissenschaftlichen-nachwuchs_node.html], the E-Prom projects looked at the influence of the doctoral phase on the careers of young scientists in the life sciences using the example of doctoral graduates from biological and medical faculties. Well over a thousand doctoral graduates (N=1796) from Bavaria, North Rhine-Westphalia and Saxony were surveyed longitudinally at several points after their doctorate using quantitative methodology. In addition, two qualitative studies were conducted to gain a deeper understanding of the doctoral phase and career entry and to shed light on the situation of advanced postdocs in their further career planning (cf. [12]). Furthermore, document analyses were used to compare doctoral requirements and regulations of almost one hundred (N=98) programmes or institutions in the three German states involved. 

The data gathered covered an extensive range of topics, from fundamental doctoral details, supervisory characteristics, activities during the doctoral phase, integration within the scientific community, and more. In the context of this project report, our aim is to provide an overview of the core findings of the project, complementing those extensively discussed in other publications.

Various activities and competencies are pivotal for academic success [18]. However, during the doctoral phase, which is this project's focal point, research emerges as paramount. Thus, within the E-Prom projects, a significant emphasis was placed on this aspect. For instance, the project's data revealed that doctoral researchers allocated a substantial portion of their time to research activities (exceeding 80% of their time), while teaching, in contrast, consumed a mere average of 10% [9]. Further, research prowess stands out as the most influential factor in securing a professorship (e.g. [18], [23] [28]). Consequently, the E-Prom projects paid particular attention to research-related self-efficacy expectations – essentially, the confidence in one’s capability to successfully undertake and complete future research tasks (refer also to [20]).

What follows is a concise summary of the E-Prom projects’ outcomes, emphasizing the doctoral phase and the initial transition to the postdoc stage.

## Results

### Individual and structured doctorates

First of all, our analyses show that formalised learning opportunities are now offered and perceived in the context of both structured and individual doctorates [24]. The results underline that a dichotomous view is not sufficient when analysing forms of doctorates. Moreover, the differences between individual and structured doctoral researchers in the life sciences are on the whole more minor than one might assume. This is due to the fact that individual doctoral researchers also have institutional structures and have to fulfil specific requirements, and that structured programmes are implemented and lived differently by individual doctoral researchers and supervisors [21], [24]. 

A comparison of the forms of doctoral studies reveals differences in particular in access to doctoral studies. Formalised recruitment and selection procedures occur more frequently in the context of structured programmes, although informal admission processes apparently still exist in practice [21]. Furthermore, supervision agreements occur somewhat more frequently in structured programmes (almost 64 percent vs. 53 percent in individual doctorates). According to the respondents, 30 percent of these agreements include concrete milestones and a work plan. Another study also showed that written goals/plans with supervisors have a significant positive influence on the success of postdocs, e.g. in terms of publication output and subjectively perceived success [5]. However, as our data show that only 30 percent of respondents in structured programmes meet regularly with supervisors, there is still a strong need for change at this point. 

Overall, the concrete design of doctoral programmes varies greatly [25]. This is evident not only in supervision arrangements, but also in the degree of internationalisation and interdisciplinarity. In addition, the range of topics covered by the courses offered varies greatly, with aspects not specifically related to research, such as career planning outside academia, rarely offered despite their high relevance for doctoral students. Approaches to avoid bias in the assessment of dissertations, for example by including external reviewers or excluding supervisors, are also hardly found in doctoral programmes to date. There is thus still a need for action on these points.

Structured programmes have a positive effect on a central variable of our research project, the so-called research-related self-efficacy expectancy [20]: this effect is partly mediated by attending courses and other learning formats, so that the voluntarily participating individual doctoral researchers (see above) could also benefit. However, the positive effect of participation in a structured programme on research-related self-efficacy expectancy remains significant even when such course attendance is statistically taken into account [24]. In addition, doctoral graduates from structured programmes took part in more courses on average than individual doctoral graduates, especially those intended to prepare them for an academic career after the doctorate [21]. In structured programmes, 54 percent took part in such courses here, while in individual doctorates it was only 15 percent of the respondents. A differentiated overview of participation in individual course formats can be found in Lachmann et al. 2020 ([21] p.15). 

### Social embedding: scientific community and supervision 

With regard to social aspects of the doctorate, our results indicate that being embedded in the scientific community is particularly important. At the time immediately after the doctorate, this was positively related to the desire to pursue a scientific career among female doctoral graduates in biology. For male doctoral graduates, there was a direct correlation between integration into the scientific community and the number of publications as first author during the doctorate. Involvement in the local working group, on the other hand, was associated neither with publication output nor with the desire to pursue a scientific career (cf. [9]) 

In the course of the qualitative interviews, it emerged that negatively experienced supervision relationships can have an impact not only on the doctoral process, but also on career aspirations and decisions after the doctorate. For example, when supervisors badmouth their doctoral researchers in front of others ([10], 144 ff). Conversely, positive effects of autonomy support on the research-related self-efficacy expectations of doctoral researchers have already been shown [28]. In a longitudinal view, however, the importance of an autonomy- and competence-supporting research environment during the doctorate recedes into the background, while good integration into the scientific communities also has a positive long-term effect on scientific career aspirations. The formation and maintenance of identification as a scientist plays a central mediating role [27]. Our data show that social integration is related to the enjoyment of research-related activities [26]. Furthermore, we found that a research environment perceived as autonomy- and competence-supportive is associated with less frustration and more enjoyment in research-related activities, and that researchers in leadership positions experience significantly more enjoyment in research than employees [26]. In addition, a positive relationship with the supervising professor and embeddedness in the scientific community was significantly associated with postdocs’ scientific career aspirations, and negatively associated with their intention to leave their current position [7]. In terms of integration into the scientific community, it is also evident that although scientific collaborations in the life sciences can have their “pitfalls”, they are evaluated positively overall and the advantages outweigh the disadvantages [6]. 

### Gender and socio-demographic characteristics 

The data from the E-Prom projects suggest that gender exerts a significant influence on scientific performance during and after the doctorate in both biological subjects and human medicine. In both subject groups, women publish significantly less than men during the doctorate (cf. [8], [9], [10]). 

Larger gender differences were found in human medicine: research-related self-efficacy expectations and the intention to pursue an academic career were significantly lower in the female sample – even under statistical control of publications and doctoral grade. This could not be explained by any of the variables surveyed and should be pursued in further research projects. 

Remarkably, only a small influence of socioeconomic background (occupation and educational background of both parents) on doctoral success was found in our samples [22]. A positive correlation was found between socioeconomic background and doctoral publications, but there were no significant correlations with grade or subjectively perceived doctoral success. It should be noted, however, that the sample considered already had a very homogeneous, high socioeconomic background and, in addition, only successfully completed doctorates were considered (cf. [22]). 

### Other personal characteristics relevant to success 

Some personal psychometric characteristics of the doctoral researchers and doctoral graduates proved to be important in the context of our results. With regard to doctoral enrolment, we find that intrinsic motives for the doctorate due to interest in research during and after the doctorate are positively and significantly correlated with the desire to pursue an academic career as well as research-related self-efficacy expectations (cf. [8], [10]). However, intrinsic motives do not seem to have an effect on the number of publications (cf. [9]). 

Research-related self-efficacy expectations proved to be the central variable [20]. This correlated significantly and strongly with the desire to pursue an academic career, controlling for the doctoral grade and publications (cf. [8], [10]). In addition, this variable also correlated significantly with the grade of the doctorate and the number of publications resulting from it [10], [20]. Since these variables were surveyed in a cross-section after the doctorate, the direction of the correlation is unclear, but a reciprocal influence is plausible (cf. [10], [20]). 

### Human medicine as a special case? 

The doctorate in medicine differs structurally significantly from the doctorate in other subjects, as it has so far often or essentially been completed alongside studies and without prior scientific experience (such as qualifying theses). In addition, the doctorate in medicine can almost be described as a standard degree, since the majority of physicians complete a doctorate – for the most part, however, this is not associated with an interest in a scientific career (summarized in [3], [10]).

In terms of personal psychometric traits, our investigations reveal that medical doctoral graduates, when compared to their counterparts from biological faculties, generally possess lower research-related self-efficacy expectations [8], [10], [20]. They are less intrinsically motivated to engage in research as the primary reason for pursuing a doctorate [10], [20], and they are also less likely to envision a scientific career post-doctorate [8], [10]. Consequently, it’s not unexpected to find that medical doctoral graduates publish fewer research articles during their doctoral studies (see [8], [10]).

Insights from our qualitative analysis, along with data from a Bavarian graduate study [https://www.bap.ihf.bayern.de/medibas], imply that these observed variances in doctoral outcomes stem from the nature of scientific training and the prevailing interests of students during their studies and doctorate (see also [6]).

However, it’s pivotal to highlight that the majority of our observations found the aforementioned patterns to be consistent across both medicine and biology. This suggests that modifying factors like self-efficacy and intrinsic motivation might enhance the appeal of research careers in medicine. The methods to instigate these positive shifts might differ by discipline, given that distinct personality traits play a role in both subject selection and study place allocation (refer to, for instance, [4], [14]). Encouraging students to gain hands-on research experience during their studies might be an effective strategy to cultivate a passion for research, even within medical courses (as outlined in [10]).

## Discussion

In conclusion, the findings from the E-Prom project emphasize the significance of research-related self-efficacy expectations and their determinants. Factors such as quality of supervision, sense of accomplishment and the emotions linked to it, social integration, structured learning experiences, as well as specific disciplines and gender, all exert influence. These elements subsequently shape the interest in pursuing an academic career. Furthermore, as one might anticipate, the inclination towards such a career is often a pivotal motivation behind the decision to embark on a doctoral journey.

From a methodological point of view, our analyses must be viewed against the background that only successfully completed doctorates were included. Thus, it was not possible to analyse the reasons for doctoral drop-outs or to make comparisons between different forms of doctorates in this regard. In addition, purely dichotomous observations were made in subject-comparative analyses, i.e. medicine and biology were compared. However, we are well aware that these subject areas are also further subdivided and in some cases differ greatly from one another. Moreover, in some areas, research in the life sciences is very interdisciplinary, so that joint work between medicine and biology is often necessary for scientific progress. Doctoral graduates from biological faculties in particular also have more diverse backgrounds and specialisations in terms of their undergraduate academic qualifications, which can certainly have an impact on the doctoral process and professional career. In the context of the E-Prom projects presented, these could not be taken into account due to low case numbers in the respective subdisciplines. 

What implications do our project's findings have for the training of emerging life scientists in Germany? In relation to structured doctoral programmes, our data suggests that while they offer a valuable avenue for doctoral training, their potential has not been consistently maximized. Moving forward, there needs to be a more in-depth scrutiny of these programmes’ structural guidelines, especially concerning how they are actively utilized by doctoral researchers, supervisors, and other stakeholders. Emphasis should be particularly placed on offerings that bolster self-efficacy, as well as on interactions within research groups and the broader scientific community. Only with such retrospective evaluations can we gauge the efficacy of these structural components and refine them for future enhancement.

Particularly in the field of medicine with its structural peculiarities in doctoral studies, which go hand in hand with little previous scientific experience on the part of doctoral students, structured programmes can be used to impart competences in scientific work. 

Furthermore, our analyses confirm that the integration of young scientists into the scientific community is beneficial in terms of career aspirations, positioning, as well as scientific results [9], [13], [15], [17], [30], [31]. This is an aspect that should be taken into account by supervisors in the context of both structured and individual doctorates. 

Likewise, our results suggest that female junior researchers should be more strongly supported. Unfortunately, the data do not provide any strong explanatory factors for their disadvantage, but greater awareness of the importance of, for example, co-authorships in the context of doctoral studies (cf. [11]) by all those involved seems sensible and appropriate.

The research-related self-efficacy expectation emerges in our data as a characteristic that is strongly associated with an objectively successful doctorate (e.g. with publications) as well as with aspirations to remain in science in the future. Thus, this construct can be helpful in the future monitoring of doctoral researchers in the context of the doctoral process or can already be used in the selection of doctoral researchers. In our view, it is particularly important to investigate and understand such personal characteristics, access and supportive framework conditions of doctoral studies: doctoral studies represent a considerable investment of time and monetary resources for those concerned and for society (cf. e.g. [1], [2], [32], [33], [34]). Transparency in selection procedures and discipline-specific guidelines with regard to quality standards of doctorates can help to train young scientists in a more targeted manner. 

## Conclusion and outlook

The findings from our project highlight the determinants that shape success during the initial stages of a scientific career and consequently influence the decision to continue in academia post-doctorate. Given that doctoral candidates are primarily tasked with demonstrating their aptitude for independent research, our project predominantly concentrated on research and activities closely linked to it. Future studies should explore how other factors, like an interest in teaching, impact subsequent career trajectories, both in terms of succeeding in academia or choosing to depart from it.

## Competing interests

The authors declare that they have no competing interests. 
